# Variation in Mutation Spectra Among CRISPR/Cas9 Mutagenized Poplars

**DOI:** 10.3389/fpls.2018.00594

**Published:** 2018-05-07

**Authors:** Estefania Elorriaga, Amy L. Klocko, Cathleen Ma, Steven H. Strauss

**Affiliations:** ^1^Department of Forest Ecosystems and Society, Oregon State University, Corvallis, OR, United States; ^2^Department of Biology, University of Colorado Colorado Springs, Colorado Springs, CO, United States

**Keywords:** *Populus*, CRISPR/Cas9, site-directed-mutagenesis, *LEAFY*, *AGAMOUS*

## Abstract

In an effort to produce reliably contained transgenic trees, we used the CRISPR/Cas9 system to alter three genes expected to be required for normal flowering in poplar (genus *Populus*). We designed synthetic guide RNAs (sgRNAs) to target the poplar homolog of the floral meristem identity gene, *LEAFY* (*LFY*), and the two poplar orthologs of the floral organ identity gene *AGAMOUS* (*AG*). We generated 557 transgenic events with sgRNA(s) and the Cas9 transgene and 49 events with Cas9 but no sgRNA, and analyzed all events by Sanger Sequencing of both alleles. Out of the 684 amplicons from events with sgRNAs, 474 had mutations in both alleles (77.5%). We sequenced both *AG* paralogs for 71 events in INRA clone 717-1B4 and 22 events in INRA clone 353-53, and found that 67 (94.4%) and 21 (95.5%) were double locus knockouts. Due partly to a single nucleotide polymorphism (SNP) present in the target region, one sgRNA targeting the *AG* paralogs was found to be completely inactive by itself (0%) but showed some activity in generating deletions when used in a construct with a second sgRNA (10.3–24.5%). Small insertion/deletion (indel) mutations were prevalent among mutated alleles of events with only one sgRNA (ranging from 94.3 to 99.1%), while large deletions were prevalent among alleles with two active sgRNAs (mean proportion of mutated alleles was 22.6% for small indels vs. 77.4% for large indels). For both *LFY* and *AG*, each individual sgRNA-gene combination had a unique mutation spectrum (*p* < 0.001). An *AG*-sgRNA construct with two sgRNAs had similar mutation spectra among two poplar clones (*p* > 0.05), however, a *LFY*-sgRNA construct with a single sgRNA gave significantly different mutation spectra among the same two clones (*p* < 0.001). The 49 empty vector control events had no mutations in either allele, and 310 potential “off-target” sequences also had no mutations in 58 transgenic events studied. CRISPR/Cas9 is a very powerful and precise system for generating loss-of-function mutations in poplars, and should be effective for generating reliably infertile trees that may promote regulatory, market, or public acceptance of genetic engineering technology.

## Introduction

Demand for forest products is expected to increase considerably with the projected population growth in the next few decades (FAO et al., 2012). We harvest forest products from wild and cultivated forests, yet clearing of wild forests comes at a high cost to natural ecosystems (Gamfeldt et al., 2013; Pimm et al., 2014). Meanwhile, plantation forests provide more timber per area than natural forests and provide some of the same ecosystem services as wild forests (Brockerhoff et al., 2008). Plantation forests only comprise 5% of the forested land but they provide about 35% of the world's forest products (FAO, 2010). Based on numerous field studies, it appears that wood yield from intensively grown plantation forests could be improved by the use of genetic engineering (GE) techniques (Strauss et al., 2017), and may be particularly important given the rapid growth of biotic and abiotic stresses on forests (Strauss et al., 2015). GE may thus lessen the effects that human demand is causing to wild forests and their ecosystems (Strauss et al., 2017). Unfortunately, regulatory and market obstacles greatly limit the ability to use GE methods, even for field research, in many parts of the world, and concerns over gene flow and resulting adventitious presence are major reasons for these obstacles. A reliable genetic containment system might be a key, enabling tool for many applications.

Site-directed mutagenesis has not been readily available in vascular plants, as in other organisms including yeast, *Drosophila*, mouse and human cells, until the advent of site specific nucleases (Weinthal et al., 2010; Voytas, 2013; Chen and Gao, 2014). The Clustered Regularly Interspaced Short Palindromic Repeats (CRISPR)/Cas gene editing system is revolutionizing reverse genetics studies in all systems including trees (Belhaj et al., 2015; Montenegro, 2016; Quétier, 2016; Song et al., 2016). It has made site-directed mutagenesis attractive and attainable in plants because of its relatively low cost, ease of use compared to other methods such as ZFNs and TALENs, and its high mutagenesis efficiency (Samanta et al., 2016; Demirci et al., 2017), including in poplar (*Populus* species) (Fan et al., 2015; Zhou et al., 2015). It should therefore enable the directed mutation of genes essential for sexual fertility—many of which are known from studies in *Arabidopsis* and other model plant species—potentially enabling the production of predictably and reliably sterile trees (reviewed in Brunner et al., 2007; Vining et al., 2012). Because intensively grown plantation forest trees such as poplar are often vegetatively propagated, and seed as well as pollen dispersal are of concern in most tree species, we chose two types of gene targets whose loss of function is expected to give bisexual sterility.

We targeted the poplar homologs of two genes essential to flower formation and morphology*, LEAFY* (*LFY*) and *AGAMOUS* (*AG*). Flowers form on the edge of shoot apical meristems (SAMs) because of the action of the meristem identity genes *LFY, APETALA 1 (AP1)*, and *CAULIFLOWER (CAL)* (Parcy, 2005; Diggle et al., 2011)*. LFY* encodes a transcription factor that regulates the expression of floral organ identity genes. The precise spatial and temporal expression of the floral organ identity genes determines the generation of the flower and is largely explained by the ABCDE model (previously known as the ABC model) (Coen and Meyerowitz, 1991; Mendoza et al., 1999; Rijpkema et al., 2010). *AG* is a class C gene that encodes a MADS box transcription factor essential for stamen, carpel, and ovule formation (Theissen et al., 2000; Krizek and Fletcher, 2005).

Strong homozygous *LFY* mutants in *Arabidopsis* are completely male sterile, and their female fertility is significantly reduced (Schultz and Haughn, 1991; Weigel et al., 1992). Homozygous *FLORICULA* (ortholog of *LFY*) mutants in snapdragon and homozygous *FALSIFLORA* (ortholog of *LFY*) mutants in tomato show complete sexual sterility (Coen et al., 1990; Molinero-Rosales et al., 1999). The *LFY* homolog in poplar, *PLFY*, is a single copy gene that shows strong expression in developing inflorescences and weak expression in vegetative tissues (Rottmann et al., 2000). Targeting of poplar *LFY* by RNA interference (RNAi) led to female trees with completely sterile flowers and apparently normal growth in the field (Klocko et al., 2016).

Homozygous *AG* mutants in *Arabidopsis* completely lose their third and fourth whorl identities, and also lose determinacy of the floral meristem (Bowman et al., 1989). Due to a relatively recent partial genome duplication, there are two *AG* orthologs in poplar, *PAG1* and *PAG2*, located on two different chromosomes (Brunner et al., 2000). They both have a similar expression pattern to that of *AG* in *Arabidopsis* and they share 89% amino acid identity with each other. Strong RNA suppression of both *AG* genes and *AG-like11* leads to healthy trees with completely sterile flowers in a field trial (Lu et al., 2018).

We designed four sgRNAs to test the mutagenesis efficiency of the CRISPR/Cas9 nuclease system by targeting the poplar orthologs to *LFY* and *AG*. We created six plant-expression plasmids; four expressing the sgRNAs individually and two expressing them in pairs, and transformed them along with a Cas9-only control vector. We were successful at generating hundreds of transgenic events with altered gene sequences. We report that the CRISPR/Cas9 system is highly efficient in generating floral gene knock-outs in poplar, and can be readily used to generate large as well as small deletions that should stably destroy protein function.

## Materials and methods

### Plant materials

Leaf, stem, and petiole explants from *in vitro* grown hybrid poplar, INRA clone 717-1B4 (female, *Populus tremula* × *P. alba*; hereafter 717) and INRA 353-38 (male, *P. tremula* × *P. tremuloides*; hereafter 353), which have been grown in our lab for numerous transgenic studies (e.g., Strauss et al., 2004; Zhang et al., 2010), were used for *Agrobacterium*-mediated plant transformation. Both clones, abbreviated as 717 and 353, were re-established from field grown material into sterile culture in 2012.

### Target gene sequencing

Partial sequencing of the *LFY* ortholog, *PLFY* (GenBank accession number U93196, Potri.015G106900), and two *AG* paralogs, *PAG1* and *PAG2* (GenBank accession numbers AF052570 and AF052571, Potri.004G064300 and Potri.011G075800) (Brunner et al., 2000; Rottmann et al., 2000), in 717 and 353 was done previously (Lu et al., 2016). For this study, further sequencing of all genes was done to find natural allelic variants outside of the target region (gene sequence between both target sites) to certify that both alleles for each gene were amplified by PCR (Table S1). Several amplicons covering the promoter region, the first exon, the first intron, and part of the second exon in *PLFY* were sequenced with various pairs of primers (Table S2). Most of the first exon in both *PAG* genes was amplified with several PCR reactions (Tables S1, S2).

### CRISPR/Cas9 target site selection

**Figure 1:**
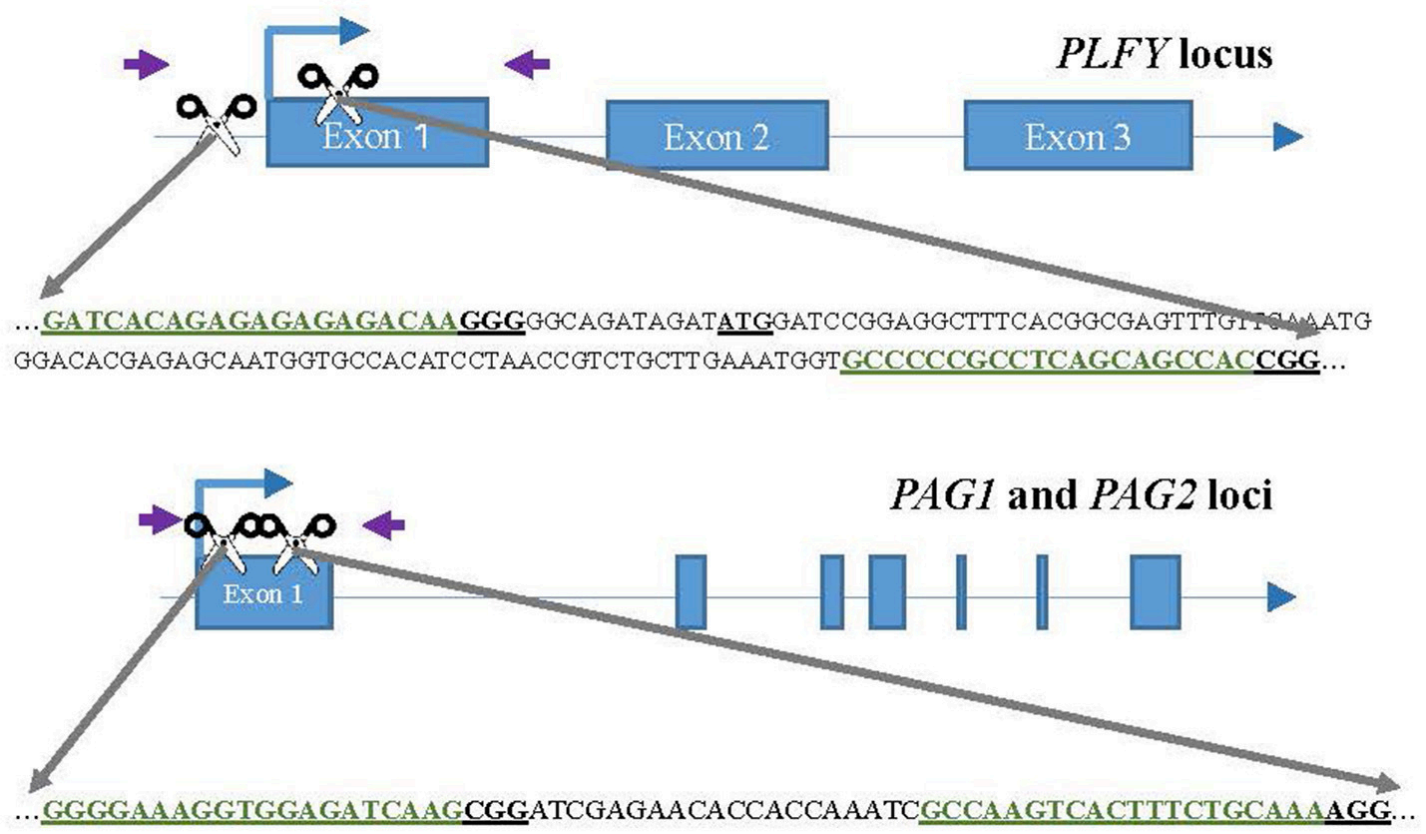
CRISPR/Cas9 sgRNA design and mutation detection in *LFY* and *AG* paralogs. Schematic representations of the target sites and the PCR assay for Sanger Sequencing. Exons and introns are represented by blue boxes and blue lines, respectively. The scissors indicate the target sites for each CRISPR/Cas9 nuclease. The purple arrows indicate the approximate location of the primers for sequencing. The target sites are colored in green inside the partial gene sequence. The underlined ATG in *LFY* indicates the location of the translation start codon.

We chose two different target sites for each gene (Figure 1), *PLFY, PAG1*, and *PAG2*, with the help of the sgRNA design online tool ZiFit (Sander et al., 2007, 2010; Hwang et al., 2013; Mali et al., 2013). The same target sites were selected for *PAG1* and *PAG2* to allow for dual gene targeting. Based on the partial sequence we had for each gene (Lu et al., 2016), we selected highly conserved sites that had no known sequence variants. However, we renewed plant material before this study in 2012 and discovered a SNP in the *PAG2* gene that was not detected in previous work.

For each target gene, we chose one target site either in the promoter region or at the beginning of the coding region, and the second target site tens to hundreds of bases 3′ in the first exon (Figure 1). The purpose was to choose targets far enough from each other to create a large deletion when both sgRNAs were present. The target sites selected had a “G” as their first base to function as the RNA polymerase start site and where followed by “NRG” given *Streptococcus pyogenes* Cas9 preference for that sequence as the Protospacer Adjacent Motif (PAM).

### CRISPR/Cas9 construct assembly

**Figure 2:**
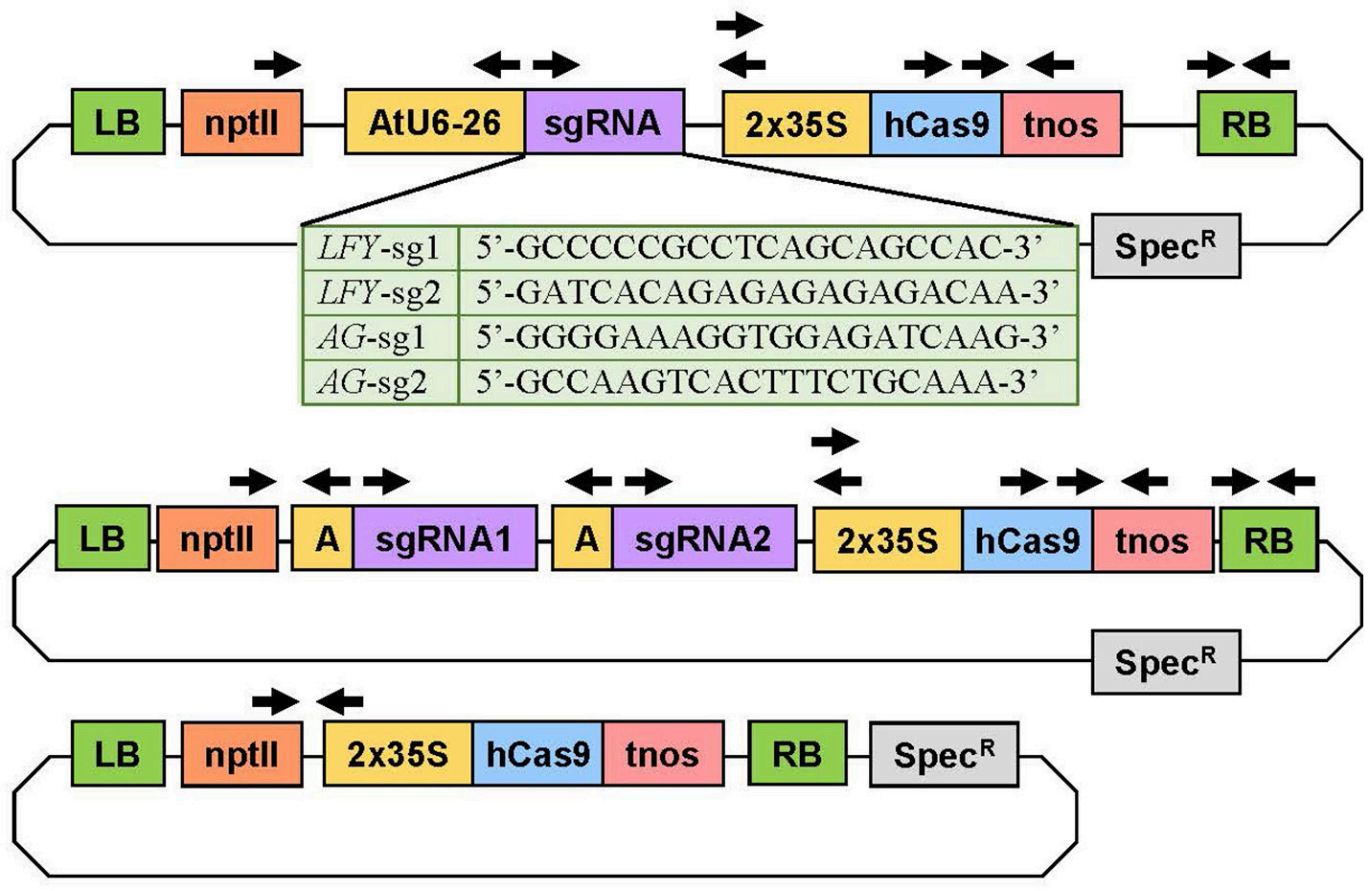
Experimental constructs targeting one or two loci simultaneously. The construct at the top was used to target a single site in the target gene(s). The table below shows the specific sequence of each sgRNA. The plasmid in the middle was used to target two loci in the same gene(s). The plasmid on bottom was the Cas9 control plasmid with no sgRNA. The arrows indicate the primers used to verify the genetic sequence of the plasmids and to determine if the independent insertion events were transgenic. 2X35S, double *Cauliflower mosaic virus* (CaMV) 35S gene promoter; AtU6-26 or A, *Arabidopsis thaliana* U6-26 gene promoter; hCas9, human codon-optimized Cas9 gene sequence from *Streptococcus pyogenes*; LB, left T-DNA border; nptII, neomycin phosphotransferase II gene sequence for kanamycin resistance; RB, right T-DNA border; sgRNA, gene-specific sgRNA sequence; Spec, spectinomycin resistance gene sequence; tnos, termination region of the nopalene synthetase gene from *Agrobacterium tumefaciens*.

To implement the CRISPR/Cas9 system in *Populus*, we selected vectors (AtU6-26SK and 35S-Cas9-SK) that had previously been proven highly active in *Arabidopsis* (Feng et al., 2013). We chose a double 35S promoter to drive the Cas9 to guarantee high expression and a human-codon optimized Cas9 because it is shown to be highly efficient in plants (Belhaj et al., 2013). We assembled seven CRISPR/Cas9 constructs; three to target *PLFY*, three to target both *PAGs* genes, and an empty-vector control for expression of Cas9 in the absence of sgRNAs (Figure 2). Out of each of the three constructs targeting a specific gene or genes, two constructs contained only one sgRNA and the last construct had both sgRNAs together. The AtU6-26SK and 35S-Cas9-SK intermediary vectors were used to assemble all the CRISPR/Cas9 constructs (Feng et al., 2013). Final constructs were assembled as previously described (Feng et al., 2013). In brief, two single-stranded 24 bp oligos were purchased from IDT (Coralville, IA, USA) for each sgRNA, where oligo 1 was of the form: bases “GATT” followed by 20 bases matching the target site and oligo 2 was of the form: bases “AAAC” followed by 20 bps matching the reverse complement of the target site. Each pair of oligos corresponding to a sgRNA was phosphorylated and annealed together in a reaction using T4 Polynucleotide Kinase (T4 PNK, NEB BioLabs, Beverly, MA) and an oligo concentration of 100 μM (thermocycler parameters: 37°C for 30 min, 95°C for 5 min, then ramp down to 25°C by decreasing 5°C every minute). The AtU6-26SK was then digested with BbsI (NEB). Each pair of annealed oligos was ligated into the digested AtU6-26SK vector using T4 ligase (NEB). For the construct with two sgRNAs, the AtU6-26SK vector with the second sgRNA was used as template in a PCR reaction (Mullis et al., 1986) and the section containing the promoter, the sgRNA, and the terminator was amplified with primers (IDT) containing 5′-KpnI and 3′-EcoRI sites. The PCR amplicon and the AtU6-26SK vector with the first sgRNA were digested with KpnI-HF (NEB) and EcoRI-HF (NEB) and ligated together using T4 ligase (NEB). Next, the promoter, sgRNA, and terminator cassettes (with one or two sgRNAs) in the modified AtU6-26SK vectors and the 35S-Cas9-SK vector were digested with HindIII (NEB) and ligated together using T4 ligase (NEB). Then, the plant expression vector pK2GW7 was digested with KpnI-HF (NEB) and ZraI (NEB). The entire piece containing the sgRNA expression cassette(s) and the Cas9 expression cassette in the modified 35S-Cas9-SK vector was digested with KpnI-HF (NEB) and SmaI (NEB) and ligated into the KpnI and ZraI sites in the already digested pK2GW7 using T4 ligase (NEB). For the empty-vector control construct, the Cas9 cassette was digested using KpnI-HF (NEB) and SmaI (NEB) from the 35S-Cas9-SK vector and ligated into the pK2GW7 already digested with KpnI and ZraI with T4 ligase (NEB). All restriction enzyme digestions were incubated for 1 h at 37°C. After incubation each digestion reaction was run on a 1% agarose gel, extracted, and purified using the Zymoclean Gel DNA Recovery kit (Zymo Research). All ligation reactions were incubated at 16°C for 12 h. After each ligation, in house-made DH5α *Escherichia coli* cells were transformed, plated in antibiotic *Luria-Bertani* media with agar (Bertani, 1951), and grown overnight for further cloning.

### *Agrobacterium*-mediated transformation

pK2GW7 constructs with CRISPR/Cas9 cassettes (one or two sgRNAs and the Cas9 gene sequence) were transformed into *Agrobacterium tumefaciens* AGL1 using the freeze and thaw method (Weigel and Glazebrook, 2006). Each CRISPR/Cas9 construct was transformed into hybrid poplar using standard methods (Filichkin et al., 2006). In brief, leaf, petiole, and stem explants from 353 and 717 *in vitro* grown plants were cocultivated with each strain of AGL1 (containing one CRISPR/Cas9 construct) for 48 h in callus induction media (CIM) in the dark. Following this, the explants were washed and then moved to CIM with antibiotic for 3 weeks of culture in the dark. After significant calli could be seen with the naked eye, the explants were moved to shoot induction media with antibiotic for 6–8 weeks, subculturing at 3- to 4-week intervals. After shoots became visible, explants were moved to shoot elongation media with antibiotic for 2–3 weeks. Last, shoots were moved to rooting media with antibiotic for 3–4 weeks. Individual transgenic events were confirmed at this point and further micropropagated.

### DNA isolation and transgene confirmation

Shoot tip and leaf tissue from *in vitro* propagated 717 and 353 individual shoots were harvested for genomic DNA extraction according to Crowley et al. (2003). Genomic DNA concentration and purity for some of the events was determined using a Nanodrop 2000 spectrophotometer (www.nanodrop.com). The presence of the transgene was verified using PCR (Mullis et al., 1986) with Econotaq DNA Polymerase (Lucigen, Middleton, Wisconsin, USA) and two sets of primers (IDT); one set near the left T-DNA border (AtU626_F1 and sgRNA_R1, Table S2), and another set near the right T-DNA border (Cas9_end_F2 and tnos_R2) (Figure 2, Table S2).

### Mutation identification

We used PCR (Mullis et al., 1986) to amplify the genomic region flanking all of the target sites. We amplified the promoter and the entire first exon in *PLFY* in order to identify as many mutation types as possible. The farthest forward and reverse primers were 229 bp upstream of *LFY*-sg2 and 333 bp downstream of *LFY*-sg1, respectively (LFY_seq_F7 and LFY_R2; product size 702 bp). For *PAG1* and *PAG2*, we amplified most of the first exon from both genes. In *PAG1*, our forward primer was 73 bp upstream of *AG*-sg1 and 138 bp downstream of *AG*-sg2 (AG1_seq_F1 and AG1_seq_R4; product size 323 bp). In *PAG2*, our forward primer was 81 bp upstream of *AG*-sg1 and 344 bp downstream of *AG*-sg2 (AG2_seq_F1 and AG2_seq_R5; product size 529 bp). Individual amplicons from each transgenic event were run on agarose gels. Bands were excised using a clean razor and DNA was extracted using the QIAEX II Gel Extraction kit (Qiagen, Hilden, Germany) or the Zymoclean Gel DNA Recovery kit (Zymo Research) following the manufacturer's instructions. The pairs of primers used for sequencing *PLFY* were LFY_seq_F1 or LFY_seq_F7 and LFY_R2 (Table S2). The primers used for sequencing *PAG1* were AG_seq_F1 or AG1_seq_F1 and AG1_seq_R4 (Table S2). The primers used for sequencing *PAG2* were AG2_seq_F1 and AG2_seq_R5. The primers used for allelic-specific PCR when sequencing *PAG1* in clone 717 were AG1I_F1 (allele one) or AG1II_F2 (allele two) and AG1_seq_R4. The primers used for allelic-specific PCR when sequencing *PAG2* in clone 717 were AG2_seq_F1 and AG2I_R4 (allele one) or AG2II_R4 (allele two). The primers used for allelic-specific PCR when sequencing *PAG1* in clone 353 were AG1I_353_F1 (allele one) or AG1II_353_F1 (allele two) and AG1_seq_R4. The primers used for allelic-specific PCR when sequencing *PAG2* in clone 353 were AG2_seq_F1 and AG2I_353_R2 (allele one) or AG2II_353_R2 (allele two). The sequence of each purified PCR product was defined using Sanger Sequencing by the Center for Genome Research and Biocomputing (CGRB) at Oregon State University. Individual sequences were aligned to the wild type (WT) sequences using MEGA6 (Tamura et al., 2013). Partial amino acid sequences were translated using MEGA6 to determine the severity of the mutation on the predicted final peptide sequence (Figure S1).

### Haplotype validation

We identified six natural SNP variants in *PLFY* in 717, two in *PAG1*, and eight in *PAG2* (Table S1). The two haplotypes are CGCTTG and TATCGA for *PLFY*, AG, and GA for *PAG1*, and AATGCCCT and GCCATTTC for *PAG2*. For clone 353, we identified five SNP variants in *PLFY*, one in *PAG1*, and five in *PAG2* (Table S1). In clone 353, the two haplotypes are ATTCC and GCCTT for *PLFY*, A and C for *PAG1*, and CATGT and AGCTA for *PAG2*. We used these SNP variants and the haplotypes they defined to ensure that both alleles had been amplified for each target gene.

### Allele characterization

We started our analysis of mutations by simultaneously amplifying both alleles of our insertion events in each PCR product. Given that most of the events with guide RNAs had different genotypes on each allele, our trace files showed double peaks. Initially to obtain an approximate ratio between biallelic (two altered alleles) and heterozygous (one altered allele and one WT allele) events, we amplified the promoter and first exon of *PLFY* for a randomly selected group of events, subcloned the allele-specific amplicons into pCR4-TOPO vector (www.invitrogen.com), and transformed DH5alpha *E. coli* cells. We included a few randomly selected homozygous mutants to certify that both alleles indeed had the same mutation. The separation of alleles allowed us to determine the specific natural haplotypes of WT 717. We also used TOPO cloning to determine the sequences of the alleles of *PAG2* for a selected group of events that were homozygous mutants in *PAG1*. We amplified both alleles simultaneously for all of our empty-vector control events because we did not expect to have different genotypes at each allele.

As we found that many events had different alleles, we utilized the online tool DSDecode (Liu et al., 2015) to genotype events with chromatograms that showed heterozygous sequences. The ab1 file with the sequence information for each event and the WT sequence of the corresponding gene were uploaded to the DSDecode online tool. Last, results were manually confirmed by locating the double peaks in the ab1 files and by ensuring that the cleavage sites were in the target regions of the sgRNAs.

For a quarter (27.9%) of our transgenic events, we used allele-specific PCR (Newton et al., 1989; Cha et al., 1992) to identify the mutations in both alleles in both *PAG1* and *PAG2*. Allele-specific primers were designed based on the natural allelic variants in each allele (Table S1).

### Characterization of mutation spectra

We compared mutation types with a prevalence higher or equal to 4.5% in most gene-sgRNA combinations (i.e., *LFY*-sg1, *LFY*-sg2, *AG1*-sg1, and *AG2*-sg2) using Pearson's Chi Square Test of Independence to test for equality of proportions (Table S6). We also employed the same test to determine if the same gene-double sgRNA combination (i.e., *LFY*-sg1sg2, *AG1*-sg1sg2, and *AG2*-sg1sg2) had the same profile in both hybrid clones (Tables S6–S8). All analyses were performed in R 3.4.1 (R Core Team, 2017) using the chisq.test function from the MASS package (Venables and Ripley, 2002). Monte Carlo simulation of 2,000 replicates were done when the sample sizes were < 100. When referring to small indel mutations, we summed the number of small deletions and small insertions.

We used the Probe Search from the sPta717 Genome (Xue et al., 2015; Zhou et al., 2015) and the Cas-OFFinder online algorithm (Bae et al., 2014) to identify genes that contained putative off-target sites in their coding region and had two or less mismatches when compared to the “seed section” of the target site (last 12 bps of the sgRNA sequence) (Sternberg et al., 2015) (Table S12). We selected two genes with off-target sites that matched 17 and 16 of the 20 bases in *LFY*-sg1 and three genes with sites that all matched 17 out of the 20 bases in *AG*-sg2. The genes that partly match *LFY*-sg1 were Potri.001G254500 and Potri.009G049600 and matched all but 2 bp in the seed sequence and all but 3 and 4 bp in the entire sgRNA sequence, respectively. The three genes that partly matched *AG*-sg2 were Potri.005G156900, Potri.013G104900, and Potri.019G077200, and they had only two mismatches in the seed region and three mismatches in the entire 20 bp sequence.

Potri.001G25450/Potri.009G049600 and Potri.013G104900/ Potri.019G077200 are pairs of paralogs and share 88.8% and 93.8% of amino acid similarity with each other, respectively. Potri.001G254500 and Potri.009G049600 encode proteins similar to *Arabidopsis UBIQUITIN-CONJUGATING ENZYME 19* (*UBC19*) and *UBIQUITIN-CONJUGATING ENZYME 20* (*UBC20*). Potri.013G104900 and Potri.019G077200 encode a MADS box transcription factor homologous to *SEEDSTICK* (*STK*, also known as *AGL11*, gene id *At4g09960*) in *Arabidopsis*. Potri.005G156900 encodes for *UBIQUITIN CARBOXYL-TERMINAL HYDROLASE 36/42* (*USP36*) similar to *UBIQUITIN-SPECIFIC PROTEASE 16* in *Arabidopsis*. None of the off-target sites had allelic variants in the sgRNA target sites (i.e., natural SNPs). We sequenced 19 events that had mutations in *PLFY* and 39 events that had mutations in *PAG1* and *PAG2;* plants were sampled for DNA extraction after 4–10 months of *in vitro* propagation. Between three to five PCR products were isolated together from gel using either the QIAEX II Gel Extraction kit (Qiagen) or the Zymoclean Gel DNA Recovery kit (Zymo Research). Sequences were defined by the Sanger Sequencing service at the CGRB. To estimate maximum off-target rates, we calculated the rates as 1/(N-alleles), and then the standard error using binomial expectation of: square root[(pq)/(2N)].

## Results

### High knockout rates in *PLFY*

Poplars have a single gene that is homologous to Arabidopsis' LFY gene. For analysis of the first guide RNA in the *PLFY* gene (*LFY*-sg1), out of 114 sequenced independent events, 103 had mutations in at least one allele and 90 events had both alleles defined by sequencing (Table 1). Out of the 90 defined events, 15 had the same mutations in both alleles (homozygous mutants), 54 had a different mutation in each allele (biallelic mutants), two were chimeric with three mutant alleles observed, eight had one mutated allele and one WT allele (heterozygous mutants), and the remaining 11 had two WT alleles (Table 1). In summary, 71 of 114 independent events had all alleles altered making the potential total knockout rate 62.3%.

**Table 1:** Numbers of mutants and rates of mutagenesis according to target gene, sgRNA, and clone.

**Gene-sgRNA**	**Clone**	**Total events (*N*)**	**Events w/both alleles defined (*N*)**	**Events with all alleles altered**			**Events with one or more WT alleles**		
				**Homoz. (A_1_/A_1_)**	**Bi-allele (A_1_/A_2_)**	**Chimera (A_1_/A_2_/A_3_)**	**Chimera (A_1_/A_2_/W)**	**Heteroz. (A_1_/W)**	**WT (W/W)**
*LFY*-sg1	717	114	90	15 (13.2%)	**54 (47.4%)**	2 (1.8%)	0 (0.0%)	8 (7.0%)	11 (9.6%)
*LFY*-sg2		45	38	12 (26.7%)	**22 (48.9%)**	0 (0.0%)	0 (0.0%)	1 (2.2%)	3 (6.7%)
*LFY*-sg1sg2		87	73	6 (6.9%)	**58 (66.7%)**	3 (3.4%)	3 (3.4%)	1 (1.1%)	2 (2.3%)
*AG1*-sg1		64	64	0 (0.0%)	0 (0.0%)	0 (0.0%)	0 (0.0%)	0 (0.0%)	**64 (100.0%)**
*AG2*-sg1		8	8	0 (0.0%)	0 (0.0%)	0 (0.0%)	0 (0.0%)	0 (0.0%)	**8 (100.0%)**
*AG1*-sg2		61	59	6 (9.8%)	**48 (78.7%)**	0 (0.0%)	0 (0.0%)	2 (3.3%)	3 (4.9%)
*AG2*-sg2		64	59	6 (9.4%)	**47 (73.4%)**	1 (1.6%)	0 (0.0%)	1 (1.6%)	3 (4.7%)
*AG1*-sg1sg2		118	89	8 (6.8%)	**67 (56.8%)**	0 (0.0%)	1 (0.8%)	3 (2.5%)	10 (8.5%)
*AG2*-sg1sg2		24	20	2 (8.3%)	**13 (54.2%)**	2 (8.3%)	0 (0.0%)	1 (4.2%)	2 (8.3%)
*LFY*-sg1sg2	353	33	26	7 (21.2%)	**15 (45.5%)**	1 (3.0%)	0 (0.0%)	0 (0.0%)	3 (9.1%)
*AG1*-sg1sg2		31	30	1 (3.2%)	**25 (80.6%)**	0 (0.0%)	0 (0.0%)	0 (0.0%)	4 (12.9%)
*AG2*-sg1sg2		35	35	4 (11.4%)	**26 (74.3%)**	0 (0.0%)	0 (0.0%)	1 (2.9%)	4 (11.4%)
Total		684	591	67 (9.8%)	**375 (54.8%)**	9 (1.3%)	4 (0.6%)	18 (2.6%)	117 (17.1%)
Total (w/out *AG*-sg1)		612	519	67 (10.9%)	**375 (61.3%)**	9 (1.5%)	4 (0.7%)	18 (2.9%)	45 (7.4%)

*The events with all alleles defined were used to calculate mutation rates and to separate events according to putative phenotype (knock-out or WT). We considered an event to be a putative “knock-out” in none of its alleles had WT sequence and “WT” if one or more of its alleles had WT sequence. A “chimera” with all its alleles altered had three mutated alleles (A1/A2/A3) and it was considered a putative knockout. A chimera with two mutated alleles and one WT allele (A1/A2/W) was considered to have WT phenotype. A, altered allele; Heteroz., heterozygote; Homoz., homozygote; W, WT allele. Different numbers in the subscript of the alleles stand for distinct alleles. The number and percentage of the most prevalent genotype for each CRISPR/Cas9 nuclease has been bolded*.

For analysis of the second guide RNA in the *PLFY* gene (*LFY*-sg2), out of 45 sequenced independent events, 42 had mutations in at least one allele and 38 had both alleles defined (Table 1). Out of the 38 defined events, 12 were homozygous mutants, 22 were biallelic mutants, one was a heterozygous mutant, and three had no mutations on both alleles (Table 1). Given the location of *LFY*-sg2 in the promoter region and all of the mutations being small indels, we did not expect to get any knockout phenotypes in this group.

We generated transgenic independent events with two sgRNAs targeting *PLFY* (*LFY*-sg1sg2) in both 717 and 353 hybrid clones. For analysis in 717, we generated 87 independent events and found 84 had mutations in at least one allele and 73 that had both alleles defined by sequencing (Table 1). Out of the 73 defined events, six were homozygotes, 58 were bi-allelic mutants, three were chimeric with all altered alleles, three were WT chimeras (two mutated alleles and a third WT allele), one was a heterozygote, and three had two WT alleles (Table 1). Thus, there were 67 of 87 independent events with both alleles altered and the putative knockout rate was 77.0%.

For analysis of *LFY*-sg1sg2 in 353, we sequenced 33 transgenic events, 30 had at least one allele mutated and 26 had both alleles defined by sequencing (Table 1). Out of the 26 events, seven were homozygous mutants, 15 were biallelic mutants, one was a chimera with all altered alleles, and three had two WT alleles (Table 1), summing to 23 of 33 independent events with altered alleles and a putative knockout rate of 69.7%.

### High double knockout rates in *PAG* genes

Poplars have two orthologous genes to *Arabidopsis' AG* gene. The second *PAG* gene was generated during a recent partial genome duplication that happened between 35 and 18 million years ago (MYA) (Tuskan et al., 2006). Thus, we were simultaneously targeting four gene copies with two sgRNAs. For analysis of the first guide RNA in *PAG1*, (*AG1*-sg1), we sequenced 64 independent transgenic events and none of them had any mutations (Table 1). For analysis of the same guide RNA in the *PAG2* gene (*AG2*-sg1), we sequenced eight of the 64 independent transgenic events from the *AG1*-sg1 group and saw no mutations (Table 1). In summary, from the analysis of the sg1 guide RNA in both *PAG* genes (*AG1*-sg1 and *AG2*-sg1), no events with altered alleles were found and the putative knockout rate was 0.0%.

For analysis of the second guide RNA in the *PAG1* gene (*AG1*-sg2), we sequenced 61 events, and 58 had mutations in at least one allele and 59 had both alleles defined by sequencing (Table 1). Out of the 59 events, six were homozygous mutants, 48 were biallelic mutants, two were heterozygous mutants, and three had no mutations in either allele (Table 1), equating to 54 of 61 independent events with altered alleles and a putative knockout rate of 88.5%. For analysis of the second guide RNA in the *PAG2* gene (*AG2*-sg2), we sequenced 64 events (61 events with *PAG1* sequenced plus three more); 61 had mutations in at least one allele and 59 had both alleles defined (Table 1). Out of the 59 events, six were homozygous mutants, 47 were biallelic mutants, one was a chimera with all altered alleles, one was a heterozygous mutant, and four had no mutations in either allele (Table 1), equating to 54 events with altered alleles and a putative knockout rate of 84.4%. Out of the 64 events with *AG*-sg2 for which we sequenced *PAG2*, two had only one allele defined (both mutations) and 52 had both alleles altered in *PAG1*. Thus, 52 (81.3%) of 64 events were putative double knockouts in *PAG1* and *PAG2*.

We also generated transgenic independent events with two sgRNAs targeting both *PAG1* and *PAG2* in 717 and 353. For analysis of PAG1 in 717, we generated 118 independent events and found that 103 of them appeared to have mutations in at least one allele; in 89 of these both alleles were defined by sequencing (Table 1). Out of the 89 defined events, eight were homozygotes, 67 were bi-alleles, one was a WT chimera, three were heterozygotes, and 10 had two WT alleles (Table 1), totaling 75 of 118 independent events with altered alleles and a putative knockout rate of 63.6%. For analysis of *PAG2* in 717, we sequenced 24 (out of the 118 we sequenced for *AG1*-sg1sg2) transgenic events; 22 had mutations in at least one allele and 20 had both alleles defined (Table 1). Out of the 20 defined events, two were homozygotes, 13 were bi-alleles, two were chimeras with all altered alleles, one was a heterozygote, and two had no mutation in either allele (Table 1), summing to 17 of 24 events with alleles altered and a putative knockout rate of 70.8%. Out of the 24 events with *AG*-sg1sg2 for which we sequenced *PAG2*, one had only one allele amplified in *PAG1*, one had both WT alleles, and 15 were putative knockouts. Therefore, 15 (62.5%) of 24 events were putative double knockouts in *PAG1* and *PAG2*.

For analysis of *PAG1* in 353, we sequenced 31 transgenic events, 27 had at least one allele mutated and 30 had both alleles defined by sequencing (Table 1). Out of the 30 events, one was a homozygote, 25 were biallelic mutants, and four had two WT alleles (Table 1), totaling 26 of 31 events with both copies altered and a putative knockout rate of 83.9%. For analysis of *PAG2* in 353, we sequenced 35 transgenic events and all of them had both alleles defined (Table 1). Out of the 35 events, four were homozygous mutants, 26 were biallelic mutants, one was a WT chimera, and four had two WT alleles (Table 1), summing to 30 of 35 events with altered alleles and a putative knockout rate of 85.7%. Out of the 30 events with both alleles altered in *PAG2*, 22 were sequenced in *PAG1*, of which one had only one allele defined and 21 had all four gene copies altered making the putative double knockout rate 95.5%.

### No mutations detected in Cas9-only transgenic controls

A total of 49 empty vector control events that had only the Cas9 gene sequence had no mutations in both alleles of *PLFY, PAG1*, and *PAG2* (totaling 294 different gene amplicons) (Table S3). Out of the 49 independent events, 32 were in 717-1B4 and 17 were in 353-53 (Table S3).

### Mutation types correspond to activity and number of sgRNAs

**Figure 3:**
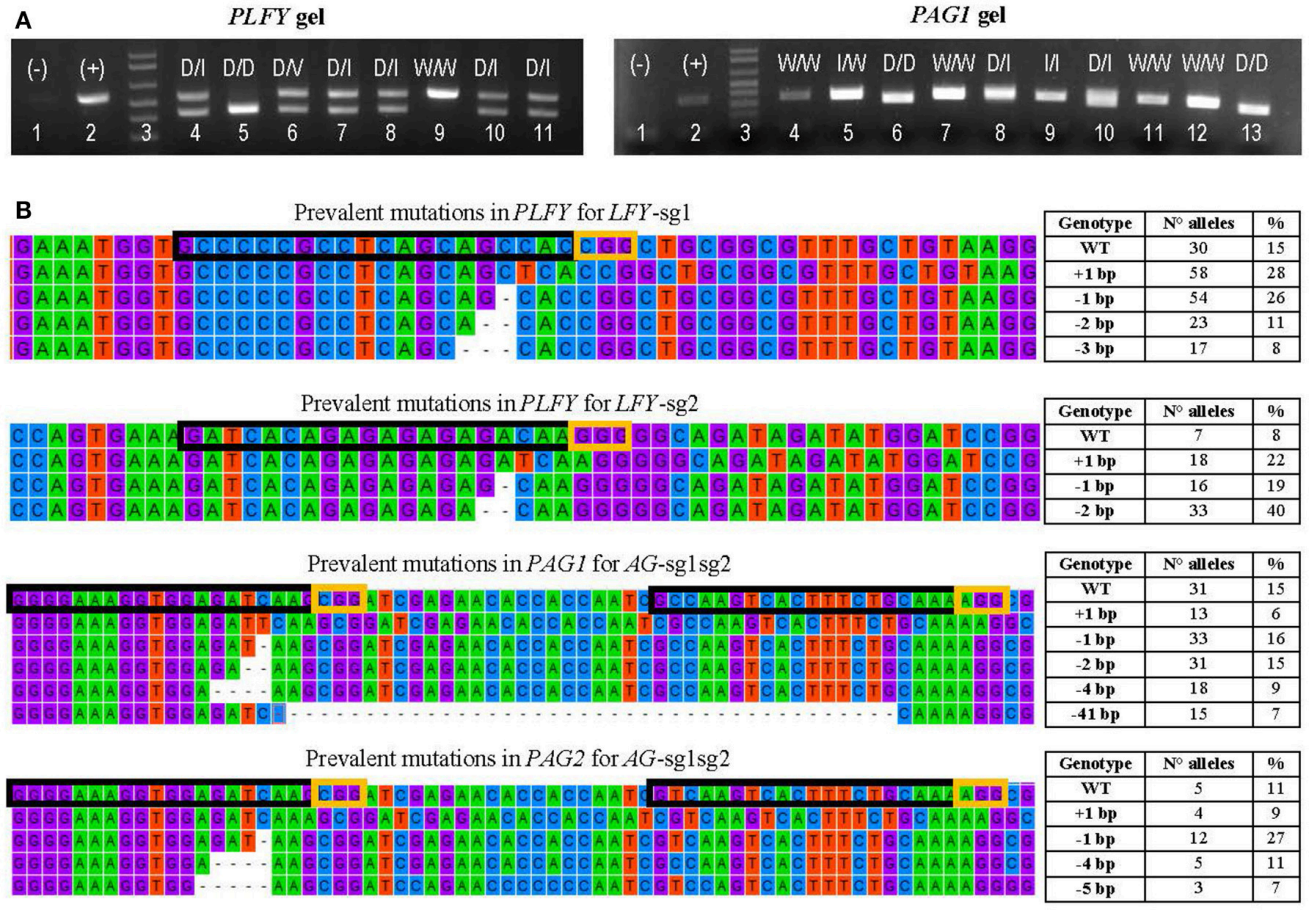
Transformation event genotyping of *LFY* and *AG* paralogs. **(A)** Example of gels from *PCRs* of *PLFY* and *PAG1* for insertion events with two sgRNAs. Symbols above each lane indicate the sequencing results of the DNA band(s). (+), positive control (–), negative control D, large deletion (16 or more base pairs); I, indel (insertion or deletion of 14 or fewer base pairs); V, inversion; W, wildtype. **(B)** Examples of the mutation types seen in alleles from mutants with one sgRNA in *PLFY* and two sgRNAs in *PAG1* and *PAG2*. The top alignment shows the partial gene sequence of *PLFY* flanking *LFY*-sg1 in the coding region. The second from the top alignment shows the partial sequence of *PLFY* flanking *LFY*-sg2 in the promoter region. The third from the top alignment shows the partial sequence of *PAG1* between *AG*-sg2 and *AG*-sg1. The bottom alignment shows the partial sequence of *PAG2* between *AG*-sg2 and *AG*-sg1. The protospacer sequence (i.e., target site) is surrounded by a black box. The PAM sites are surrounded by a yellow box. The dashes indicate deleted base pairs. The tables on the right indicate the mutation seen in each row, the number of alleles with that mutation, and the percentage that the number represents in each group.

Events generated with one active sgRNA had mostly small deletions (60.9–79.5%, Table 2) and secondly small insertions (17.0–33.3%, Table 2). Meanwhile, events with two active sgRNAs targeting the same gene (i.e., *LFY*-sg1sg2) had mainly large deletions (64.1–90.7% in 717 and 353, respectively, Table 2, Figure 3A) and secondly small indel mutations (5.6–30.1% in 353 and 717, respectively, Table 2). Events with both sgRNAs targeting *PAGs* had mostly small indels (75.5–86.9%, Table 2) but large deletions were also seen (10.3–24.5%, Table 2). Events with a SNP in their target did not have any mutations (i.e., *AG1*-sg1 and *AG2*-sg1, Table 2).

**Table 2:** Mutation types.

**Gene-sgRNA**	**Clone**	**Alleles defined (*N*)**	**Mutation in each allele**							
			**Small deletion**	**Small insertion**	**Small subs**.	**Large deletion**	**Large insertion**	**Invers**.	**Large subs**.	**Undef**.
*LFY*-sg1	717	174	**106 (60.9%)**	*58 (33.3%)*	0 (0.0%)	2 (1.1%)	5 (2.9%)	0 (0.0%)	0 (0.0%)	3 (1.7%)
*LFY*-sg2		76	**53 (69.7%)**	*20 (26.3%)*	1 (1.3%)	1 (1.3%)	0 (0.0%)	0 (0.0%)	0 (0.0%)	1 (1.3%)
*LFY*-sg1sg2		153	*31 (20.3%)*	15 (9.8%)	0 (0.0%)	**98 (64.1%)**	0 (0.0%)	8 (5.2%)	0 (0.0%)	1 (0.7%)
*AG1*-sg1		64	0 (0.0%)	0 (0.0%)	0 (0.0%)	0 (0.0%)	0 (0.0%)	0 (0.0%)	0 (0.0%)	0 (0.0%)
*AG2*-sg1		8	0 (0.0%)	0 (0.0%)	0 (0.0%)	0 (0.0%)	0 (0.0%)	0 (0.0%)	0 (0.0%)	0 (0.0%)
*AG1*-sg2		112	**89 (79.5%)**	*19 (17.0%)*	0 (0.0%)	2 (1.8%)	2 (1.8%)	0 (0.0%)	0 (0.0%)	0 (0.0%)
*AG2*-sg2		116	**92 (79.3%)**	*23 (19.8%)*	0 (0.0%)	0 (0.0%)	1 (0.9%)	0 (0.0%)	0 (0.0%)	0 (0.0%)
*AG1*-sg1sg2		177	**121 (68.4%)**	15 (8.5%)	4 (2.3%)	*30 (16.9%)*	1 (0.6%)	0 (0.0%)	0 (0.0%)	6 (3.4%)
*AG2*-sg1sg2		39	**27 (69.2%)**	*4 (10.3%)*	0 (0.0%)	*4 (10.3%)*	0 (0.0%)	0 (0.0%)	0 (0.0%)	4 (10.3%)
*LFY*-sg1sg2	353	54	1 (1.9%)	*2 (3.7%)*	0 (0.0%)	**49 (90.7%)**	0 (0.0%)	1 (1.9%)	1 (1.9%)	0 (0.0%)
*AG1*-sg1sg2		53	**37 (69.8%)**	3 (5.7%)	0 (0.0%)	*13 (24.5%)*	0 (0.0%)	0 (0.0%)	0 (0.0%)	0 (0.0%)
*AG2*-sg1sg2		61	**44 (72.1%)**	*9 (14.8%)*	0 (0.0%)	7 (11.5%)	1 (1.6%)	0 (0.0%)	0 (0.0%)	0 (0.0%)
Total		1159								

*Rates of major classes of mutations from each gene-sgRNA combination. Undefined refers to insertion lines whose alleles were difficult to define by DSDecode. The most prevalent mutation type is highlighted in green and bold and the second most prevalent type in yellow and italics. Small refers to mutations of 15 bp or less. Invers., inversion; N, number; subs., substitution; Undef., undefined*.

### Mutation spectra varies among sgRNA targets

After defining 1,159 alleles in 561 events (Table 2), we suspected that there might be distinct mutation spectra for each gene-sgRNA combination (Table S4). The combinations *LFY*-sg1, *LFY*-sg2, *AG1*-sg2, and *AG2*-sg2 in 717 all had significantly different mutation spectra [χ^2^: 105.05, 15 degrees of freedom (*df*), *p* < 0.001; Table S5]. Among the 171 separate mutated alleles belonging to *LFY*-sg1, 33.9% had a 1 bp insertion, 31.6% had a 1 bp deletion, 13.6% had 2 bp deletion, 9.9% had a three bp deletion, 2.3% had a 4 bp deletion, and 8.8% had one of nine other possible mutations (Figure 3B, Table S4). Meanwhile, from the 75 alleles sequenced belonging to *LFY*-sg2, 44.0% had 2 bp deletion, 24.0% had 1 bp insertions, and 21.3% had 1 bp deletions (Figure 3B, Table S4). Among the 112 alleles belonging to *AG1*-sg2 and the 116 alleles belonging to *AG2*-sg2, most alleles had a 1 bp deletion (20.5% for in *PAG1* and 35.3% in *PAG2*) (Figure 3B, Table S4). Yet, for the rest of the alleles in *AG1*-sg2, 18.8% had a 4 bp deletion, 16.1% had a 1 bp insertion, and 12.5% had a 2 bp deletion (Table S4). Meanwhile, for the remaining alleles in *AG2*-sg2, 18.1% had a 1 bp insertion, 14.7% had a 4 bp deletion, and 8.6% had a 3 bp deletion (Table S4). Nonetheless, the spectrum from *AG1*-sg2 is not significantly different from that of *AG2*-sg2 (χ^2^: 8.15, 5 *df*, *p* > 0.05) (Table S5). All other pair comparisons of mutation spectra differed significantly (*p* < 0.001, Table S5).

Given the difference in activity between *LFY*-sg1sg2 and either *AG1*-sg1sg2 or *AG2*-sg1sg2, we did not consider it meaningful to compare their mutation spectra. Nonetheless, we decided to compare the mutation spectrum of *LFY*-sg1sg2 in 717 and in 353 (Table S6) and the mutation spectrum of both *AG1*-sg1sg2 and *AG2*-sg1sg2 in 717 and 353 (Tables S8, S10). Events with *LFY*-sg1sg2 in 717 and in 353 had a significantly different mutation spectrum (*p* < 0.001, Table S7). Meanwhile, 717 and 353 events with either *AG1*-sg1sg2 or *AG2*-sg1sg2 did not have significantly different mutation spectra (*p* >> 0.05, Tables S9, S11).

### Absence of mutations detected in off-target sites

A concern in using site-directed mutagenesis is the possibility of off-target mutations. We identified two potential off-site target sites that were similar to the target sites of *PLFY*, and three that were similar to the target sites of the *PAG* genes (Table S12). We selected events for analysis in which the desired target sites were mutated, indicative of a functional CRISPR/Cas9 locus. In total, we genotyped 310 alleles for off-target mutations, but saw no mutations in any of these sequences. Specifically, we found no mutations in either allele of 19 transgenic events with mutations in *PLFY* in both of the selected genes, and also saw no mutations in either allele of the 39 transgenic events with mutation in the selected *PAG1* off-target genes (Table S12). Thus, the off-target mutation rate is expected to be less than about 5% for the *PLFY* off-targets (2.6 ± 1.8%) and less than about 2% for the *PAG1* off-targets (1.3 ± 0.9%).

## Discussion

The purpose of this work was to examine the mutagenesis efficiency and pattern produced by CRISPR/Cas9 nucleases directed at endogenous floral genes of poplar. Because poplars are naturally outcrossing species with high levels of heterozygosity, it was essential to characterize both alleles at each locus using allele-specific primers or by cloning and sequencing PCR products using conserved primer sites. Initially, we amplified both alleles together, and used the DSDecode software to analyze difficult heterozygous samples (Ma et al., 2016). However, for 717 events with *AG*-sg2 and the 353 events with *AG*-sg1sg2, we amplified and sequenced separate alleles using allele-specific primers for both *PAG1* and *PAG2*. A few mutated lines had both alleles amplified together that were difficult to genotype with certainty by DSDecode, and we labeled them as “undefined” (Table 2).

A minor goal of this research work was to determine the prevalence of off-target mutations. We did not detect any mutations in 155 amplicons from specific loci (total of 310 alleles), corresponding to five off-target sites. These potential targets were similar to either of our *PLFY* or *PAG* target sites, differing in only three or four bases out of 20 base pairs of the sgRNA. The events surveyed, which included the entire CRISPR/Cas9 locus, had been growing in Magenta boxes for 6–12 months, and subcultured every 2–3 months, before tissue was sampled for DNA isolation, providing ample time for mutagenesis. A lack of off-target mutagenesis has been reported in many CRISPR/Cas studies in plants (*Arabidopsis thaliana, N. benthamiana*, hybrid poplar, rice, soybean, sweet orange, and wheat) with up to seven mismatches (Lawrenson et al., 2015; Sauer et al., 2016; Schiml and Puchta, 2016; Wolt et al., 2016). They have also not been detected in three genome scale studies (Feng et al., 2013; Peterson et al., 2016). However, off-target mutagenesis has been detected in a few plant studies, with rates ranging from 1.6 to 13.0% with one or two mismatches in the last 12bp of the sgRNA (Xie and Yang, 2013; Jacobs et al., 2015; Lawrenson et al., 2015; Sauer et al., 2016) and with rates ranging between 1.6 and 9.7% with one to three mismatches in the first eight bp (Upadhyay et al., 2013; Zhang et al., 2014; Xu et al., 2015). One case that is of interest found mutations in T1 rice plants that had constitutive Cas9 and sgRNA expression, similar to our own studies (Xu et al., 2015). Clearly, off-target rates appear to be low, but additional studies are needed, especially in systems such as trees where CRISPR/Cas9 expression may continue for many months or even years.

No mutations were seen in either allele of the three target genes, *PLFY, PAG1*, and *PAG2*, in 49 empty vector control events that were transformed with the Cas9 gene sequence but no sgRNA. Thus, as expected the CRISPR/Cas9 system requires both a nuclease and fully functional RNA components for specific mutagenesis, and shows that somaclonal varation associated with *in vitro* culture and *Agrobacterium* transformation had a negligible influence. Given our large sample size, we were able to characterize mutations according to type for each sgRNA. The specific class of mutation seen depended on the number of sgRNAs present in the binary vector. As in other plant studies, most of the events with one active sgRNA had small deletions or single base insertions (reviewed by Bortesi et al., 2016). Meanwhile, lines with two active sgRNAs targeting the same gene, i.e., *LFY*-sg1sg2, had mainly large deletions (between 64.1 and 90.7%) removing the DNA between the sites, many indels (between 5.6 and 30.1%), and some inversions (between 1.9 and 5.2%). This is the third study on CRISPR/Cas9 plants that reports inversions. Large deletions and inversions have also been reported in *Arabidopsis* (Zhang et al., 2017) and rice (Zhou et al., 2014; Liang et al., 2016) when using two sgRNAs separated between 200 bp or 245 kb. However, our independent events transformed with two sgRNAs that were not of comparable activity, i.e., *AG*-sg1sg2, had mainly small deletions like those lines transformed with only one sgRNA.

The most common peptide modifications expected from translating the altered alleles with only one sgRNA (i.e., *LFY*-sg1 and *AG*-sg2) or two sgRNAs with one inactive (i.e., *AG*-sg1sg2) included removal of essential amino acids (see −3 bp deletion with *LFY*-sg1 in Figure S1), early stop codons, and frame-shifted proteins (Figure S1). We occasionally saw insertions leading to predicted peptides with extra amino acids (data not shown). We did not translate the peptide sequence for *LFY*-sg2 because this sgRNA targeted the promoter, so we do not expect it to modify the *PLFY* protein sequence. With two active sgRNAs, we mainly predicted truncated or frame-shift proteins.

In this study, we characterized a large number of events (684) and alleles (1,159) by direct Sanger Sequencing. From this data, we noticed that most of the gene-sgRNA combinations had a unique mutation spectrum, suggesting that their distinct sequences or the adjacent chromosome region affect the character of the resulting mutations. van Overbeek et al. (2016) first described such an effect in a study done on 223 CRISPR/Cas9 target sites within human cells. They found that the specific mutation seen for each target sequence were likely due to the local adjacent sequence and not due to the guide RNA sequence *per se* or the genomic region.

Another goal was to select sgRNAs that would be able to induce mutations in more than one gene to get a complete loss-of-function mutant. For *PAG* we needed to alter four gene copies, the two alleles of *PAG1* and the two alleles of *PAG2*, as these two *AG*-like genes appear to share protein function (Brunner et al., 2000). Successful multi-gene targeting has been previously documented in pig, mouse, and moss (Wang et al., 2013; Yang et al., 2015; Lopez-Obando et al., 2016). The sgRNA *AG*-sg2 had high mutation rates in both *PAG1* and *PAG2*, generating several potential complete *PAG* loss-of-function (i.e., double putative knockout) mutants. Out of 54 events transformed with *AG*-sg2 with both *PAG1* and *PAG2* defined, 52 of 64 (81.3%) events were confirmed putative double knockouts in both *AG* genes. The *AG*-sg1sg2 sgRNA was also highly active. Out of the 24 events transformed with *AG*-sg1sg2 in clone 717 with both *PAG1* and *PAG2* defined, 15 (62.5%) were double putative knockouts. In addition, out of the 22 events transformed with *AG*-sg1sg2 in clone 353 with both *PAG1* and *PAG2* defined, 21 (95.5%) were double putative knockouts.

A major goal was to study the rate at which the system produced complete knockouts (i.e., loss-of-function) events for each of our target genes. The *AG*-sg1 nuclease however, induced no mutations in either *PAG1* or *PAG2*. This lack of mutation was likely in part due to the presence of a SNP in *PAG2* in our new 717 stock (Zhou et al., 2015), and possibly also low activity by the sgRNA. Nonetheless, when this guide RNA was present in a construct with a second, active guide RNA, we observed several deletions with an endpoint at the target of this otherwise inactive sgRNA, indicating it may have retained some level of Cas9 guide activity.

Three of the four CRISPR/Cas9 nucleases, i.e., *LFY*-sg1, *LFY*-sg2, and *AG*-sg2, generated high rates of mutagenesis in their corresponding target gene(s) when acting individually, creating many putative loss-of-function lines. Of all the events with either *LFY*-sg1 or *LFY*-sg1sg2 in 717, 62.3 and 77.0%, respectively, are putative proteins knockouts. In 353, 69.7% of the events are also putative protein knockouts, and like in 717, they had mainly truncated and/or frame-shifted proteins. Clearly, CRISPR/Cas9 is a very powerful technology that, for the first time, can readily generate loss of function mutations at single loci as well as at the paralogous gene families that are so prevalent in poplar (Tuskan et al., 2006) and many other plant species.

## Author contributions

EE: designed the study, sequenced the target and off-target genes, designed and constructed the vectors, gathered, analyzed, and interpreted the data, and wrote the manuscript; AK: helped with vector construction, study design, and writing the manuscript; CM: performed the plant stable transformation, regeneration, and selection; SS: supervised all of the work. The manuscript was read and approved by all the authors.

### Conflict of interest statement

The authors declare that the research was conducted in the absence of any commercial or financial relationships that could be construed as a potential conflict of interest.
