# Supplementary material for: Variation in Mutation Spectra Among CRISPR/Cas9 Mutagenized Poplars
Source: Front Plant Sci. 2018 May 7;9:594. doi: 10.3389/fpls.2018.00594 (PMC5949366; doi:10.3389/fpls.2018.00594)
Supplement: Supplementary file 1 [file Table_1.docx]

Table S1. Partial genetic sequence of the target genes and the off-target sites. The 20bp protospacer sequences (i.e. the target sites), the off-target sites, the transcription-starting triplet (ATG), and the SNPs are underlined and in bold. SNPs are defined as: K, G or T; M, A or C; R, A or G; Y, C or T; W, A or T.

| **Gene-clone** | **Gene sequence** |
| --- | --- |
| ***Target Loci*** | |
| *PLFY* in 717 | CTGTCCAGTTCCGAAGAAACATCAAAACCCTTTAATTCTGTTAGCTTCC**Y**AATACATACAAAAAAGAAAAAAAGACAA**R**AAACTTGTCCTGTTAAGGGCAGTTTTGGTATA**Y**AAATAAAACAAGAAGCTCACTTGTCTTTATATATCTACCAAATCCAAGACATGCACCAGTGAAA**GATCACAGAGAGAGAGACAA**GGGGGCAGATAGAT**ATG**GATCCGGAGGCTTTCACGGCGAGTTTGTTCAAATGGGACACGAGAGCAATGGTGCCACATCCTAACCGTCTGCTTGAAATGGT**GCCCCCGCCTCAGCAGCCAC**CGGCTGCGGCGTTTGCTGTAAGGCCAAGGGAGCTATGTGGGCTAGAGGAGTTGTTTCAAGCTTATGGTATTAGGTACTACACGGCAGCGAAAATAGCTGAACTCGGGTTCACAGTGAACACCCTTTTGGACATGAAAGA**Y**GAGGAGCTTGATGAAATGATGAATAGTTTGTCTCAGATCTTTAGGTGGGATCTTCTTGTTGGTGAGAGGTATGGTATTAAAGCTGCTGTTAGAGCTGAAAGAAGAAGGCTTGATGAGGAGGATCCTAGGCGTAGGCAATTGCTCTCTGGTGATAATAATACAAATACTCTTGATGCTCTCTCCCAAGAAGGTTTGGTTAGCATTGATTCTACCTTTTAGTGTAATTAAGCTAAGCTCATACTATTACTAGCTATAGGAG**K**CCATGGCCGTTTT**R** |
| *PLFY* in 353 | CTGTCCAGTTCCGAAGAAACATCAAAACCCTTTAATTCTGTTAGCTTCCCAATACATACAAAAAAGAAAAAAAGACAA**R**AAACTTGTCCTGTTAAGGGCAGTTTTGGTATA**Y**AAATAAAACAAGAAGCTCACTTGTCTTTATATATCTACCAAATCCAAGACATGCACCAGTGAAA**GATCACAGAGAGAGAGACAA**GGGGGCAGATAGAT**ATG**GATCCGGAGGCTTTCACGGCGAGTTTGTTCAAATGGGA**Y**ACGAGAGCAATGGTGCCACATCCTAACCG**Y**CTGCTTGAAATGGT**GCCCCCGCCTCAGCAGCCAC**CGGCTGCGGCGTTTGCTGTAAGGCCAAGGGAGCTATGTGGGCTAGAGGAGTTGTTTCAAGCTTATGGTATTAGGTACTACACGGCAGCGAAAATAGCTGAACTCGGGTTCACAGTGAACACCCTTTTGGACATGAAAGATGAGGAGCTTGATGAAATGATGAATAGTTTGTCTCAGATCTT**Y**AGGTGGGATCTTCTTGTTGGTGAGAGGTATGG |
| *PAG1* in 717 | GCTAGACTGCAGCT**ATG**GAATATCAAAATGAATCCCTTGAGAGCTCCCCCCTGAGGAAGCT**R**GGAA**GGGGAAAGGTGGAGATCAAG**CGGATCGAGAACACCACCAATC**GCCAAGTCACTTTCTGCAAA**AGGCGCAGTGGTTTGCTCAAGAAAGCCTACGA**R**TTATCTGTTCTTTGCGATGCTGAGGTTGCACTCATCGTCTTCTCTACCCGCGGTCGCCTTTATGAGTACTCTAACGATAGGTAAATAAATCTAATTTTAGATATATGCTTCTCTGGATCTTAAATTCTCCATGTTACAAGCCCTCT |
| *PAG1* in 353 | GCAGCT**ATG**GAATATCAAAATGAATCCCTTGAGAGCTCCCC**M**CTGAGGAAGCTGGGAA**GGGGAAAGGTGGAGATCAAG**CGGATCGAGAACACCACCAATC**GCCAAGTCACTTTCTGCAAA**AGGCGCAGTGGTTTGCTCAAGAAAGCCTACGAATTATCTGTTCTTTGCGATGCTGAGGTTGCACTCATCGTCTTCTCTACCCGCGGTCGCCTTTATGAGTACTCTAACGATAGGTAAATAAATCTAATTTTAGATATATGCTTCTCTGGATCTTAAATTCTCC |
| *PAG2* in 717 | GCTAGCAGCAGCT**ATG**GCATACCAAAATGAATCCCAAGAGAGCTCTCCCCTGAGGAAGCTGGG**R**A**GGGGAAAGGTGGAGATCAAG**CGGATCGAGAACACCAC**M**AATC**GYCAAGTCACTTTCTGCAAA**AGGCGGAATGGTTTGCTCAAGAAAGCCTATGAATTATCTGTTCTTTGCGATGCTGAGGTTGCACTCATCGTCTTCTCCAGCCGTGGACGCCTTTATGAGTACTCTAACAATAGGTATATACTTAGTTCCTC**W**GCTCATGAATTCTCCATGTTGCAA**R**CCCTCTTCAAGTGCTCACAGTTGGTTTTTCTTGCTT**Y**CTCAT**Y**CAAAGGGATTTGTTTTTT**YY**TTTT |
| *PAG2* in 353 | **ATG**GCATACCAAAATGAATCCCAAGAGAGCTCTCCCCTGAGGAAGCTGGGGA**GGGGAAAGGTGGAGATCAAG**CGGATCGAGAACACCAC**M**AATC**GCCAAGTCACTTTCTGCAAA**AGGCGGAATGGTTTGCTCAAGAAAGCCTATGAATTATCTGTTCTTTGCGATGCTGAGGTTGCACTCATCGTCTTCTCCAGCCGTGGACGCCTTTATGAGTACTCTAACAATAGGTATAT**R**CTTAGTTCCT**Y**G**K**CTCATGAATTCTCCATGTTGCAAGCCCTCTTCAAGTGCTCACAGTTGGTTTTTCTTGCTTTCTCATCCAAAGGGATTTG**W**TTTTTCTTTTTGTTTATGCCAGGGTTAATTTTTATGGTTTTT |
| ***Off-target loci*** | |
| *UBC19* in 717 (Potri.001G254500) | AGAGACAATGGCAACTGTTAATGGGTATCAAGGGAATACTCCG**GTGGCTGCTCCGGCGGGGAC**TACCCCATCAAAACAGACTGTCAC**W**GCGGCAAAGATTGTCGATACGCAATCCGTGCTTAAACGGTAATTTTCTTTTCTTTTTGCAT**Y**TGATCTGTTCTTTTCATTTGTCAAACCATGTAATATAT**MW**C**RM**G**WKW**TA**WMWSKRKW**T**W**T**W**TT**W**T**RK**G**KK**TT**Y**T**Y**TTT**KYKYRW**A**WR**T**GWKW**TT**K**TG**WSWY**TTTTTT**Y**T**YYK**T**KW**T**WWW**A**WM** |
| *UBC20* in 717 (Potri.009G049600) | TATTAATGGGGTATACTCCG**GTGGCTGCTCCGGCAGGGAC**TACCCCATCAAAACAGACTGTCCCATCGGCAAAGACTGTTGATACACAATCCGTGCTTAAACGGTATT**W**TTTTTTT**Y**T |
| *USP36* in 717 (Potri.005G156900) | TCCGCTGGGCTGTTTTATAATTTAGGTGTAAATGGCGAGGTGCGGTTG**CGAGAAAGGAGGAGATCAAG**AGGCTGTTGGTTTTGGCAGCGGAGGAAGCCGCTAGGGCTGAGTTTGAGGCCGCGGCTTCATACGGCACCGTTCCGGTGGTGACAAATAACTATCAATGTGCTGTTTGTTTTTGC**Y**CGACAACGACACGGTGTGCCCGCTGTAAAGCTGTTAGATATTGGTATGT**K**AATTTCGTGTTAATGCTTGATTTTTATCGGGTTTTAGTCCTTAATTG**M**GTTGAATTCAGGTTTG |
| *STK.1* in 717 (Potri.013G104900) | ACTGTTGCATTGCCTAGCTATTCCATGCTTTTTTTGGTAGACATGAAATGTAAAAGTCAGATAAGCTAGCTATTAGGTCAAGAAAATTGCTTGATAAGAGCATATATAATATAGAAGCTTCTTTGGGTTGTGAAAGAATTGATCTTTTGTGTAGACATGGGAA**GAGGAAAGATTGAGATCAAG**AGGATCGAGAACACTACGAATCGTCAGGTTACTTTCTGCAAGAGAAGAAATGGGCTGTTGAAGAAAGCCTATGAATTATCTGTCCTTTGTGATGCTGAAGTCTCCCTCATCGTCTTCTCCAGCCGTGGCCGTCTCTATGAGTACGCCAACAACAAGTAACTTTACCTTCCCTAAAT |
| *STK.2* in 717 (Potri.019G077200) | CTTTTTTTTGCTCTTAATTTTGTTCCCATTTTCTA{T,TTTAA}TTTACTCTTTATAAAAA**K**ATTTTTTTACCATTTACTTCTCTACAGTCTTTCTCAAACTGTTGC**R**ATTACCTTGCTATTCCATGCTTTTTTGGTAGGCATGACATGTAAAGGTCAGATCAGCTAGCTATTAGATTAAGAAAACTGGCGCATATATAAYACATAAGCTTGTATGGGTTATGAAAGAAAC**R**ATCTTTTGTGTAGACATGGGAA**GAGGAAAGATTGAGATCAAG**AGGATCGAGAACACCACCAATCGTCAGGTTACTTTCTGCAAGAGGAGAAATGGGCTCTTGAAGAAAGCTTATGAATTATCAGTTCT**K**TGTGATGCTGAAGTTGCTCTCATCGTCTTCTCTAGCCGTGGCCGTCTCTATGAGTACGCCAACAACAAGTAATTTTACCTTCTCCTT**R**TTGTCTTTTCTTTTGGATCTTGA**W**GG**R**AACCTCC{T,-}TTTCTTT |
