# Supplementary material for: Variation in Mutation Spectra Among CRISPR/Cas9 Mutagenized Poplars
Source: Front Plant Sci. 2018 May 7;9:594. doi: 10.3389/fpls.2018.00594 (PMC5949366; doi:10.3389/fpls.2018.00594)
Supplement: Supplementary file 2 [file Table_2.docx]

Table S2. Table of primers, their sequence, and their specific use.

| Primer name | Primer sequence (5’ to 3’) | Use(s) |
| --- | --- | --- |
| AtU626_F1 | CTTCAAAAGTCCCACATCGC | Verifying transgene sequence; event genotyping |
| sgRNA_R1 | GCCGCCAGTGTGATGGATA | Verifying transgene sequence; event genotyping |
| Cas9_F1 | CACGACGGAGACTACAAGGA | Verifying transgene sequence; event genotyping |
| Cas9_R1 | TCCTTGTAGTCTCCGTCGTG | Verifying transgene sequence; event genotyping |
| Cas9_mid_F1 | GTGGCCTATTCTGTGCTGGT | Verifying transgene sequence; event genotyping |
| Cas9_end_F2 | CCTACAACAAGCACCGGGAT | Verifying transgene sequence; event genotyping |
| tnos_R2 | AACGATCGGGGAAATTCGAG | Verifying transgene sequence; event genotyping |
| RB_F1 | GAAGGCGGGAAACGACAATC | Verifying transgene sequence; event genotyping |
| RB_R1 | CGGATAAACCTTTTCACGCCC | Verifying transgene sequence; event genotyping |
| LFY_seq_F1 | CCTGTTAAGGGCAGTTTTGG | Sequencing *PLFY* in both clones |
| LFY_seq_F7 | TGCAGGGAACCAAATGTGTG | Sequencing *PLFY* in both clones |
| LFY_R2 | AACCTTCTTGGGAGAGAGCA | Sequencing *PLFY* in both clones |
| AG_seq_F1 | AGTTTGTGTTTTGGATCAGC | Sequencing *PAG1* in both clones |
| AG1_seq_F1 | GTTGTCACTCAGTTTGTGTTTTGGA | Sequencing *PAG1* in both clones |
| AG1_seq_R4 | GACAGCGACCACATGC | Sequencing *PAG1* in both clones |
| AG2_seq_F1 | TGCTGTCTTCACCCAGTTTGT | Sequencing *PAG2* in both clones |
| AG2_seq_R5 | AAAACCTTGACACCAGGCTCC | Sequencing *PAG2* in both clones |
| AG1I_F2 | TCACTCAGTTTGTGTTTTGGATCAG | Sequencing allele one of *PAG1* in 717 |
| AG1II_F1 | CACTCAGTTTGTGTTTTGGATCATC | Sequencing allele two of *PAG1* in 717 |
| AG2I_R4 | TTTGCAACATGGAGAATTCATGAGCT | Sequencing allele one of *PAG2* in 717 |
| AG2II_R4 | CTTGCAACATGGAGAATTCATGAGCA | Sequencing allele two of *PAG2* in 717 |
| AG1I_353_F1 | CCCTTGAGAGCTCCCCAC | Sequencing allele one of *PAG1* in 353 |
| AG1II_353_F1 | CCTTGAGAGCTCCCCCC | Sequencing allele two of *PAG1* in 353 |
| AG2I_353_R2 | TGCAACATGGAGAATTCATGAGC | Sequencing allele one of *PAG2* in 353 |
| AG2II_353_R2 | TGCAACATGGAGAATTCATGAGA | Sequencing allele two of *PAG2* in 353 |
| P1G254500_F | TGTTGGTGCTTTCGATACCCT | Sequencing off-target Potri.001G254500 |
| P1G254500_R | ACGGTTAGATAAAGAATCAGTCACA | Sequencing off-target Potri.001G254500 |
| P9G049600_F | TGGGTTTTCTTTCTTTTGGATTCT | Sequencing off-target Potri.009G049600 |
| P9G049600_R | AGATCACAAACCACATTCATAAACA | Sequencing off-target Potri.009G049600 |
| OffAG_5F1 | TAGGGTTTTCGAGCCTGGTG | Sequencing off-target Potri.005G156900 |
| OffAG_5R1 | TCTCCCCAGAACCAAACCTGA | Sequencing off-target Potri.005G156900 |
| OffAG_13F1 | TGGAAACAGCTTTGCACTTCC | Sequencing off-target Potri.013G104900 |
| OffAG_13R1 | ATGGTATGAAGATTTAGGGAAGGT | Sequencing off-target Potri.013G104900 |
| OffAG_19F1 | AGAAACAGATTTGCACACCCT | Sequencing off-target Potri.019G077200 |
| OffAG_19R1 | AGACCTAGTGATCTGTGAGAAAGA | Sequencing off-target Potri.019G077200 |
