# Supplementary material for: Variation in Mutation Spectra Among CRISPR/Cas9 Mutagenized Poplars
Source: Front Plant Sci. 2018 May 7;9:594. doi: 10.3389/fpls.2018.00594 (PMC5949366; doi:10.3389/fpls.2018.00594)
Supplement: Supplementary file 3 [file Table_3.docx]

Table S3. Lack of mutations on target sites in empty vector controls. We sequenced the target sites corresponding to the four guide RNAs of events transformed with only the Cas9 sequence (i.e. no guide RNA) and found no mutations in both alleles of all target genes. N; number.

| Transgene | Clone | Target gene | Events (N) | Gene amplicons (N) | Mutations (rate) |
| --- | --- | --- | --- | --- | --- |
| *Cas9* only | 717 | *PLFY* | 32 | 64 | 0 (0%) |
|  |  | *PAG1* |  | 64 | 0 (0%) |
|  |  | *PAG2* |  | 64 | 0 (0%) |
|  | 353 | *PLFY* | 17 | 34 | 0 (0%) |
|  |  | *PAG1* |  | 34 | 0 (0%) |
|  |  | *PAG2* |  | 34 | 0 (0%) |
| Total | | | 49 | 294 | 0 (0%) |
