# Supplementary material for: Variation in Mutation Spectra Among CRISPR/Cas9 Mutagenized Poplars
Source: Front Plant Sci. 2018 May 7;9:594. doi: 10.3389/fpls.2018.00594 (PMC5949366; doi:10.3389/fpls.2018.00594)
Supplement: Supplementary file 4 [file Table_4.docx]

Table S4. Mutation spectra of the different gene-sgRNA combinations with only one sgRNA. The most prevalent mutation type for each specific group is in bold. The “other” mutation type refers to nine, four, ten, and eight types corresponding to *LFY-*sg1, *LFY-*sg2, *AG1-*sg2, *AG2-*sg2 respectively with lower than 4.5% prevalence. “Other” is not bolded for *AG1*-sg2 because it is made up of more than one type of mutation. bp; base pairs

|  | **1 bp insertion** | **1 bp deletion** | **2 bp deletion** | **3 bp deletion** | **4 bp deletion** | **other** | **Total** |
| --- | --- | --- | --- | --- | --- | --- | --- |
| ***LFY*-sg1** | **58**  **(33.9%)** | 54  (31.6%) | 23  (13.5%) | 17  (9.9%) | 4  (2.3%) | 15  (8.8%) | 171 |
| ***LFY*-sg2** | 18  (24.0%) | 16  (21.3%) | **33 (44.0%)** | 1  (1.3%) | 3  (4.0%) | 4  (5.3%) | 75 |
| ***AG1*-sg2** | 18  (16.1%) | **23 (20.5%)** | 14  (12.5%) | 11  (9.8%) | 21  (18.8%) | 25  (22.3%) | 112 |
| ***AG2*-sg2** | 21  (18.1%) | **41 (35.3%)** | 8  (6.9%) | 10  (8.6%) | 17  (14.7%) | 19  (16.4%) | 116 |
| **Total** | 115 (24.3%) | 134 (28.3%) | 78  (16.5%) | 39  (8.2%) | 45  (9.5%) | 63  (13.3%) | 474 |
