# Supplementary material for: Variation in Mutation Spectra Among CRISPR/Cas9 Mutagenized Poplars
Source: Front Plant Sci. 2018 May 7;9:594. doi: 10.3389/fpls.2018.00594 (PMC5949366; doi:10.3389/fpls.2018.00594)
Supplement: Supplementary file 5 [file Table_5.docx]

Table S5. Results table for the proportion comparison of all mutation spectra. Pearson’s chi-squared test of independence was used to test if the mutation signature were different between gene-sgRNA combinations.

| Mutation spectra comparison tested | X-squared | Degrees of freedom | P-value |
| --- | --- | --- | --- |
| All spectra | 105.1 | 15 | 5.0e-04 |
| *LFY-*sg1 *vs. LFY-*sg2 | 31.5 | 5 | 5.0e-04 |
| *LFY-*sg1 *vs. AG1-*sg2 | 40.5 | 5 | 1.2e-07 |
| *LFY-*sg1 *vs. AG2-*sg2 | 27.2 | 5 | 5.3e-05 |
| *LFY-*sg2 *vs. AG1-*sg2 | 40.2 | 5 | 5.0e-04 |
| *LFY-*sg2 *vs. AG2-*sg2 | 46.7 | 5 | 5.0e-04 |
| *AG1-*sg2*vs. AG2-*sg2 | 8.2 | 5 | 1.5e-01 |
