# Supplementary material for: Variation in Mutation Spectra Among CRISPR/Cas9 Mutagenized Poplars
Source: Front Plant Sci. 2018 May 7;9:594. doi: 10.3389/fpls.2018.00594 (PMC5949366; doi:10.3389/fpls.2018.00594)
Supplement: Supplementary file 6 [file Table_6.docx]

Table S6. Mutation spectra generated by the same CRISPR/Cas9 nuclease in the *PLFY* gene in two different hybrid poplar clones. The most prevalent mutation type for each specific group is in bold. The “other-large” and the “other-small” mutation types refer to 12 and 22 different mutations types respectively with lower than 4.5% prevalence. Chi-squared test of independence was used to determine if the mutation spectra are different.

|  | **120 bp deletion** | **121 bp deletion** | **120 bp inversion** | **122 bp deletion** | **other-large** | **other-small** | **Total** |
| --- | --- | --- | --- | --- | --- | --- | --- |
| ***LFY*-sg1sg2 in 717** | **61**  **(40.1%)** | 8  (5.3%) | 7  (4.6%) | 7  (4.6%) | 23 (15.1%) | 46  (30.3%) | 152 |
| ***LFY*-sg1sg2 in 353** | **32**  **(59.3%)** | 2  (3.7%) | 1  (1.9%) | 10  (18.5%) | 6  (11.1%) | 3  (5.6%) | 54 |
| **Total** | 93  (45.1%) | 10 (4.9%) | 8  (3.9%) | 17  (8.3%) | 29 (14.1%) | 49  (23.8%) | 206 |
