# Supplementary material for: Variation in Mutation Spectra Among CRISPR/Cas9 Mutagenized Poplars
Source: Front Plant Sci. 2018 May 7;9:594. doi: 10.3389/fpls.2018.00594 (PMC5949366; doi:10.3389/fpls.2018.00594)
Supplement: Supplementary file 7 [file Table_7.docx]

Table S7. Results table for the proportion comparison of the mutation spectra of *LFY-*sg1sg2 in two different poplar clones. Pearson’s chi-squared test of independence was used to test if the mutation spectra were different between the different clones.

| Mutation spectra comparison tested | X-squared | Degrees of freedom | P-value |
| --- | --- | --- | --- |
| *LFY-*sg1sg2 in 717 *vs.*  *LFY-*sg1sg2 in 353 | 24.2 | 5 | 5.0e-04 |
