# Supplementary material for: Variation in Mutation Spectra Among CRISPR/Cas9 Mutagenized Poplars
Source: Front Plant Sci. 2018 May 7;9:594. doi: 10.3389/fpls.2018.00594 (PMC5949366; doi:10.3389/fpls.2018.00594)
Supplement: Supplementary file 8 [file Table_8.docx]

Table S8. Mutation spectra generated by the same CRISPR/Cas9 nuclease in the *PAG1* gene in two different hybrid poplar clones. The most prevalent mutation type for each specific group is in bold. The “other” mutation type refers to 20 different mutation types with lower than 4.5% prevalence. “Other” is not bolded for *AG1*-sg1sg2 in 717 because it is made up of more than one type of mutation.

|  | **1 bp deletion** | **2 bp deletion** | **3 bp deletion** | **4 bp deletion** | **5 bp deletion** | **41 bp deletion** | **44 bp deletion** | **1 bp insertion** | **other** | **Total** |
| --- | --- | --- | --- | --- | --- | --- | --- | --- | --- | --- |
| ***AG1*-sg1sg2 in 717** | **33 (19.3%)** | 31 (18.1%) | 10 (5.8%) | 18 (10.5%) | 13 (7.6%) | 15 (8.8%) | 4 (2.3%) | 13 (7.6%) | 34 (19.9%) | 171 |
| ***AG1*-sg1sg2 in 353** | **14 (26.4%)** | 7 (13.2%) | 4 (7.5%) | 5 (9.4%) | 3 (5.7%) | 8 (15.1%) | 3 (5.7%) | 3  (5.7%) | 6 (11.3%) | 53 |
| **Total** | 47 | 38 | 14 | 23 | 16 | 23 | 7 | 16 | 40 | 224 |
