# Supplementary material for: Variation in Mutation Spectra Among CRISPR/Cas9 Mutagenized Poplars
Source: Front Plant Sci. 2018 May 7;9:594. doi: 10.3389/fpls.2018.00594 (PMC5949366; doi:10.3389/fpls.2018.00594)
Supplement: Supplementary file 10 [file Table_10.docx]

Table S10. Mutation spectra generated by the same CRISPR/Cas9 nuclease in the *PAG2* gene in two different hybrid poplar clones. The most prevalent mutation type for each specific group is in bold. The “other” mutation type refers to 11 different mutation types with lower than 4.5% prevalence.

|  | **1 bp deletion** | **2 bp deletion** | **3 bp deletion** | **4 bp deletion** | **5 bp deletion** | **41 bp deletion** | **1 bp insertion** | **other** | **Total** |
| --- | --- | --- | --- | --- | --- | --- | --- | --- | --- |
| ***AG2*-sg1sg2 in 717** | **12 (34.3%)** | 2  (5.7%) | 2  (5.7%) | 5  (14.3%) | 3  (8.6%) | 2  (5.7%) | 4 (11.4%) | 5 (14.3%) | 35 |
| ***AG2*-sg1sg2 in 353** | **23 (37.7%)** | 8  (13.1%) | 3  (4.9%) | 2  (3.3%) | 3  (4.9%) | 2  (3.3%) | 8 (13.1%) | 12 (19.7%) | 61 |
| **Total** | 35 | 10 | 5 | 7 | 6 | 4 | 12 | 17 | 96 |
