# Supplementary material for: Variation in Mutation Spectra Among CRISPR/Cas9 Mutagenized Poplars
Source: Front Plant Sci. 2018 May 7;9:594. doi: 10.3389/fpls.2018.00594 (PMC5949366; doi:10.3389/fpls.2018.00594)
Supplement: Supplementary file 11 [file Table_11.docx]

Table S11. Results table for the proportion comparison of the mutation spectra of *AG2-*sg1sg2 in two different poplar clones. Pearson’s chi-squared test of independence was used to test if the mutation signatures were different between the different clones.

| Mutation spectra comparison tested | X-squared | Degrees of freedom | P-value |
| --- | --- | --- | --- |
| *AG2-*sg1sg2 in 717 *vs.*  *AG2-*sg1sg2 in 353 | 6.2 | 7 | 0.5 |
