# Supplementary material for: Variation in Mutation Spectra Among CRISPR/Cas9 Mutagenized Poplars
Source: Front Plant Sci. 2018 May 7;9:594. doi: 10.3389/fpls.2018.00594 (PMC5949366; doi:10.3389/fpls.2018.00594)
Supplement: Supplementary file 12 [file Table_12.docx]

Table S12. Off-target sites studied for rate of mutagenesis. The selected targets differed by three or four bases (the bases that did not match the target are shown in lowercase). PAM sites shown in lowercase and in bold. N; number.

| Target sgRNA | Target gene | Off-target site sequence | Gene name in *Arabidopsis* | Poplar gene ID | | Mismatches (N) | Events (N) | Mutations (N) | |
| --- | --- | --- | --- | --- | --- | --- | --- | --- | --- |
| *LFY*-sg1 | *PLFY* | GtCCCCGCCggAGCAGCCAC**cgg** | Ubiquitin-conjugating enzyme 19 (UBC19) | | Potri.001G254500 | 3 | 19 | | 0 |
|  |  | GtCCCtGCCggAGCAGCCAC**cgg** | Ubiquitin-conjugating enzyme 20 (UBC20) | | Potri.009G049600 | 4 | 19 | | 0 |
| *AG*-sg2 | *PAG1*, *PAG2* | cGaGAAAGGaGGAGATCAAG**agg** | Ubiquitin-specific protease 16 (USP36) | | Potri.005G156900 | 3 | 39 | | 0 |
|  |  | GaGGAAAGaTtGAGATCAAG**agg** | *SEEDSTICK (STK)* | | Potri.013G104900 | 3 | 39 | | 0 |
|  |  | GaGGAAAGaTtGAGATCAAG**agg** | *SEEDSTICK (STK)* | | Potri.019G077200 | 3 | 39 | | 0 |
