# Supplementary material for: Variation in Mutation Spectra Among CRISPR/Cas9 Mutagenized Poplars
Source: Front Plant Sci. 2018 May 7;9:594. doi: 10.3389/fpls.2018.00594 (PMC5949366; doi:10.3389/fpls.2018.00594)
Supplement: Supplementary file 13 [file Image_1.pdf]

Amino acid modifications in *PLFY* for *LFY*-sg1

Sequence	Genotype
MDPEAFTASLFKWDTRAMVPHPNRLLEMVPPPQQPPAAAFAVRPRELCGLEELFQAYGIRYYTAA	WT
MDPEAFTASLFKWDTRAMVPHPNRLLEMVPPPQQLTGCGVCCKAKGAMWARGVVSSLWY*VLHGS	+1 bp
MDPEAFTASLFKWDTRAMVPHPNRLLEMVPPPQQHRLRRLL*GQGSYVG*RSCFKLMVLGTTRQR	-1 bp
MDPEAFTASLFKWDTRAMVPHPNRLLEMVPPPQHTGCGVCCKAKGAMWARGVVSSLWY*VLHGSE	-2 bp
MDPEAFTASLFKWDTRAMVPHPNRLLEMVPPPQPPAAAFAVRPRELCGLEELFQAYGIRYYTAAK	-3 bp

Amino acid modifications in *PLFY* for *LFY*-sg1sg2

Sequence	Genotype
MDPEAFTASLFKWDTRAMVPHPNRLLEMVPPPQQPPAAAFAVRPRELC	WT
MWHHCSRVPFEQTRRESLRIHIYLPPCTGCGVCCKAKGAMWARGVVSS	120 bp inversion
MWHHCSRVPFEQTRRESLRIHIYLPPCHRLRRLL*GQGSYVG*RSCFK	121 bp inversion
MWARGVVSSLWY*VLHGSENS*TRVHSEHPFGHER*GA**NDE*FVSD	-120 bp
MWARGVVSSLWY*VLHGSENS*TRVHSEHPFGHERRGA**NDE*FVSD	-121 bp
MK**IVCLRSLGGIFLLVRGMVLKLLLELKEEG?MRRILGVGNCSLVI	-130 bp

Amino acid modifications in *PAG1* for *AG*-sg1sg2

Sequence	Genotype
MEYQNESLESSPLRKLGRGKVEIKRIENTTNRQVTFCKRRSGLLKKAYELSVLCDAEVALIVFST	WT
MEYQNESLESSPLRKLGRGKVEIQADREHHQSPSHFLQKAQWFAQESLRIICSLRC*GCTHRLLY	+1 bp
MEYQNESLESSPLRKLGRGKVEISGSRTPPIAKSLSAKGAVVCSRKPTNYLFFAMLRLHSSSSLP	-1 bp
MEYQNESLESSPLRKLGRGKVEKADREHHQSPSHFLQKAQWFAQESLRIICSLRC*GCTHRLLYP	-2 bp
MEYQNESLESSPLRKLGRGKVESGSRTPPIAKSLSAKGAVVCSRKPTNYLFFAMLRLHSSSSLPA	-4 bp
MEYQNESLESSPLRKLGRGKVEIQKAQWFAQESLRIICSLRC*GCTHRLLYPRSPL*VL*R*VNK	-41 bp

Amino acid modifications in *PAG2* for *AG*-sg1sg2

Sequence	Genotype
MAYQNESQESSPLRKLGRGKVEIKRIENTTNRQVTFCKRRNGLLKKAYELSVLCDAEVALIVFSS	WT
MAYQNESQESSPLRKLGRGKVEIKADREHHQSSSHFLQKAEWFAQESL*IICSLRC*GCTHRLLQ	+1 bp
MAYQNESQESSPLRKLGRGKVEISGSRTPPIVKSLSAKGGMVCSRKPMNYLFFAMLRLHSSSSPA	-1 bp
MAYQNESQESSPLRKLGRGKVESGSRTPPIAKSLSAKGGMVCSRKPMNYLFFAMLRLHSSSSPAV	-4 bp
MAYQNESQESSPLRKLGRGKVEADREHHQSPSHFLQKAEWFAQESL*IICSLRC*GCTHRLLQPW	-5 bp

Fig S1. Diversity in putative amino acid modifications to the WT peptide sequence in 717. These peptide alignments are the partial translation of the sequence alignment from the most common mutations seen in events with *LFY*-sg1, *LFY*-sg1sg2, and *AG*-sg1sg2. The first line in each alignment shows the WT sequence. Stop codons are shown with a *. The tables to the right identify the specific mutation that led to the peptide modification in each row.
